# Pediatric Lyme Disease Biobank, United States, 2015–2020

**DOI:** 10.3201/eid2612.200920

**Published:** 2020-12

**Authors:** Lise E. Nigrovic, Desire N. Neville, Fran Balamuth, Michael N. Levas, Jonathan E. Bennett, Anupam B. Kharbanda, Amy D. Thompson, John A. Branda, Aris C. Garro, the Pedi Lyme Net Working Group

**Affiliations:** Boston Children's Hospital, Boston, Massachussetts, USA (L.E. Nigrovic);; Children’s Hospital of Pittsburgh, Pittsburgh, Pennsylvania, USA (D.N. Neville);; Milwaukee Children's Hospital, Milwaukee, Wisonsin, USA (F. Balamuth);; Children's Hospital of Philadelphia, Philadelphia, Pennsylvania, USA (M.N. Levas);; Nemours/Alfred I. duPont Children's Hospital, Wilmington, Delaware, USA (J.E. Bennett, A.D. Thompson);; Children’s Minnesota, Minneapolis, Minnesota, USA (A.B. Kharbanda);; Massachusetts General Hospital, Boston (J.A. Branda);; Rhode Island Hospital, Providence, Rhode Island, USA (A.C. Garro)

**Keywords:** Lyme disease, biobank, diagnosis, pediatrics, vector-borne infections, tick-borne infections, zoonoses, bacterial zoonoses, bacteria, United States, Borrelia burgdorferi

## Abstract

In 2015, we founded Pedi Lyme Net, a pediatric Lyme disease research network comprising 8 emergency departments in the United States. Of 2,497 children evaluated at 1 of these sites for Lyme disease, 515 (20.6%) were infected. This network is a unique resource for evaluating new approaches for diagnosing Lyme disease in children.

Children are disproportionally affected by Lyme disease, which is diagnosed in »300,000 persons in the United States each year (*1*). Clinicians diagnose Lyme disease using a 2-tier examination of enzyme immunoassay (EIA) and immunoblot results. Current Lyme disease diagnostic tests have well-described limitations that include false negatives early in disease (*3*) and inability to distinguish between resolved, active, and recurrent infections (*4*). Clinicians must also wait several days for Lyme disease serologic results, a delay that might contribute to late or unnecessary treatment with antimicrobial drugs. The increased incidence of Lyme disease, limitations of current tests, and lack of studies in children demonstrate the need for a systematic approach to Lyme disease diagnosis in children.

Developing improved diagnostic techniques relies on biobanks of samples collected from patients with Lyme disease and clinical mimics (i.e., patients with similar signs and symptoms caused by non-Lyme illnesses). The US Centers for Disease Control and Prevention (Atlanta, GA, USA) curated the first Lyme disease biobank with samples from 86 adults with Lyme disease, 144 clinical mimics, and 203 healthy controls from 11 collection sites (*5*). The Study of Lyme Disease Immunology and Clinical Events (http://www.slicestudies.org) at the Johns Hopkins Lyme Disease Research Center (Baltimore, MD, USA) enrolled 40 adults with an erythema migrans (EM) lesion and followed up with patients for 1 year. The Lyme Disease Biobank, supported by the Bay Area Lyme Foundation, has enrolled 550 adults with Lyme disease evaluated at 7 primary-care collection sites (*6*). To date, none of these biobanks have included children or used emergency departments for enrollment.

In 2015, we founded Pedi Lyme Net, a pediatric Lyme disease research network comprising 8 emergency departments in a diverse range of areas to which Lyme disease is endemic. We conducted a prospective cohort study of children evaluated for Lyme at 1 of of these emergency departments (Appendix Figure 1). The Pediatric Lyme Disease Biobank, housed at Boston Children’s Hospital (Boston, MA, USA), stores and distributes the biosamples collected from enrolled children (*7*).

We describe enrolled children 1–21 years of age who underwent emergency department evaluation for Lyme disease during June 1, 2015–January 31, 2020 (Table). We obtained informed consent from parents/guardians for study participation and child assent for those >8 years of age. Informed consent documents were available in English and Spanish. We defined disease stage on the basis of signs and symptoms: early (i.e., single EM lesion), early disseminated (i.e., multiple EM lesions, cranial neuritis, meningitis, or carditis) or late (i.e., arthritis or arthralgia). In addition, as asymptomatic controls, we enrolled children undergoing intravenous cannulation for procedural sedation for fracture reduction or laceration repair without acute infectious symptoms. We implemented standard operating procedures at each of the participating sites (Appendix Table). All deidentified data were collected electronically with Research Electronic Data Capture housed at Harvard University (https://catalyst.harvard.edu/services/redcap).

**Table:** Characteristics of enrolled children with Lyme disease and clinical mimics, United States, 2015–2020*

Characteristics	Lyme disease	Clinical mimics	p value
Total	515	1,982	
Demographics			
Median age, y (IQR)	8 (6–11)	9 (5–14)	0.02
Sex			
M	345 (67.0)	1,048 (52.9)	<0.01
F	170 (33.0)	934 (47.1)	
Race			
White	438 (85.0)	1,482 (74.8)	<0.01
Black	42 (8.2)	255 (12.9)	
Asian	8 (1.6)	53 (2.7)	
Pacific Islander	0	3 (0.1)	
Native American	0	2 (0.1)	
Other	22 (4.3)	156 (7.9)	
Missing data	5 (1.0)	31 (1.6)	
Ethnicity			
Hispanic	33 (6.4)	234 (11.8)	<0.01
Non-Hispanic	480 (93.2)	1,734 (87.5)	
Missing data	2 (0.4)	14 (0.7)	

History			
Presentation during peak Lyme season†	343/515 (66.6)	1,187/1,982 (59.9)	<0.01
Previous Lyme disease	47/514 (9.1)	79/1,945 (4.1)	<0.01
Tick bite within past year	73/468 (15.6)	150/1,808 (8.3)	<0.01
Fever	176/507 (34.7)	732/1,959 (37.4	0.35
Headache	152/510 (29.8)	760/1,926 (39.5)	<0.01

Examination			
Erythema migrans lesion	54 (10.5)	NA	NA
Facial palsy	59 (11.5)	158 (8.0)	<0.01
Lumbar puncture performed	47 (9.1)	155 (7.8)	0.33
Meningitis	21/47 (44.7)	57/155 (36.8)	
Arthritis (joint swelling)	286 (55.5)	556 (28.1)	<0.01

*Values are no. (%) except as indicated. NA, not applicable. †June–October.

We defined Lyme disease on the basis of an EM lesion diagnosed by the treating clinician or positive serologic results with compatible symptoms. We took serum samples from all enrolled patients, including asymptomatic controls, and conducted a C6 EIA on each sample. If the EIA results were positive or equivocal, we also conducted a Western immunoblot interpreted using standard criteria (*8*). We considered a positive IgM immunoblot paired with a negative IgG immunoblot to be positive only if symptoms lasted <30 days (*10*). We classified symptomatic children who tested negative for Lyme disease as clinical mimics. We compared characteristics of children with Lyme disease and mimics using the χ^2^ test for categorical variables and the Mann-Whitney test for continuous variables with SPSS Statistics 23.0 (IBM Corp., https://www.ibm.com).

We enrolled and obtained samples from 2,497 symptomatic and 377 asymptomatic control patients (Appendix Figure 2). Overall, 515 (20.6% of symptomatic patients) had Lyme disease; of these children, 46 (8.9%) had an EM lesion alone, 461 (89.5%) had a positive 2-tier serology alone, and 8 (1.6%) had both. Of the asymptomatic control patients, 4 (1.1%) had positive 2-tier serology.

Our Pediatric Lyme Disease Biobank is unique because it includes biosamples from pediatric patients, clinical mimics, and diverse geographic regions. The samples are linked to demographic, clinical, laboratory, and treatment data about each patient. With >2,800 children enrolled, this biobank is a unique resource for researching Lyme disease diagnosis in children.

Our biobank has a few limitations. First, we enrolled a convenience sample of children depending on the availability of study staff. However, in this study, the proportion of children with Lyme disease did not differ between enrolled and unenrolled but eligible patients. Second, some children with early or early-disseminated Lyme disease might have had false negative serologic results. However, we conducted follow-up to identify children who had initially negative 2-tier Lyme serologic results but tested positive within 30 days of enrollment. Finally, because our network includes only 8 enrollment sites, we were unable to include all regions to which Lyme disease is endemic. 

Appendix Additional information on data collected for Pediatric Lyme Disease Biobank, United States, 2015–2020.
