# Supplementary material for: Pediatric Lyme Disease Biobank, United States, 2015–2020
Source: Emerg Infect Dis. 2020 Dec;26(12):3099–101. doi: 10.3201/eid2612.200920 (PMC7706969; doi:10.3201/eid2612.200920)
Supplement: Appendix — Additional information on data collected for Pediatric Lyme Disease Biobank, United States, 2015–2020. [file 20-0920-Techapp-s1.pdf]

Article DOI: https://doi.org/10.3201/eid2612.200920

Pediatric Lyme Disease Biobank, United States, 2015–2020

Appendix Table. Variables analyzed in children evaluated for Lyme Disease, United States, 2015–2020

Variable	Subcategories
Demographics	Age, gender, race, ethnicity
History	History of previous Lyme and timing, antimicrobial pretreatment and duration, recognized tick bite, fever, headache, arthralgia
Examination	Erythema migrans lesion(s) number and size, meningismus, cranial nerve palsy, joint swelling, clinician suspicion of Lyme disease (10-point scale)
Follow-up (30 d)	Treatment received, outcome
Medical record review	
Management	Antimicrobial treatment, hospitalization, procedures performed, complications
Lyme testing (≤30 d)	Lyme serology, Lyme PCR
Laboratory studies	Complete blood cell count, C-reactive protein, erythrocyte sedimentation rate, cerebrospinal fluid results, synovial fluid results
Microbiology results	Blood, synovial, and spinal fluid cultures
Biosamples	
Samples collected	Serum, plasma, whole blood, RNA, urine
Test type	C6 enzyme-linked immunoassay, immunoblot

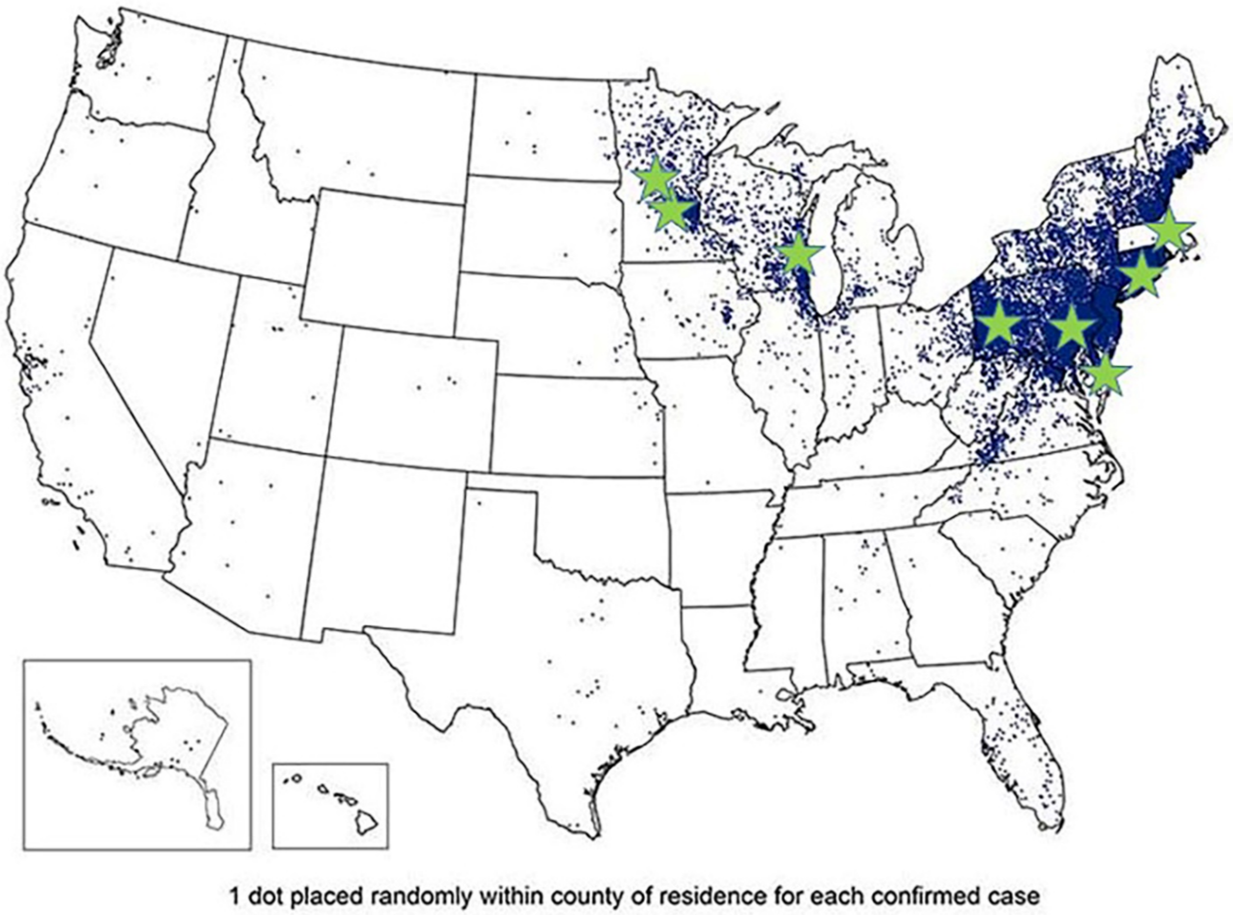

Appendix Figure 1. Pedi Lyme Net enrollment centers, United States, 2015–2020. Figure created using the 2019 Centers for Disease Control and Prevention Lyme disease map. Blue dots indicate the county of residence for every reported Lyme disease case. Green stars indicate Pedi Lyme Net 2020 enrollment centers.

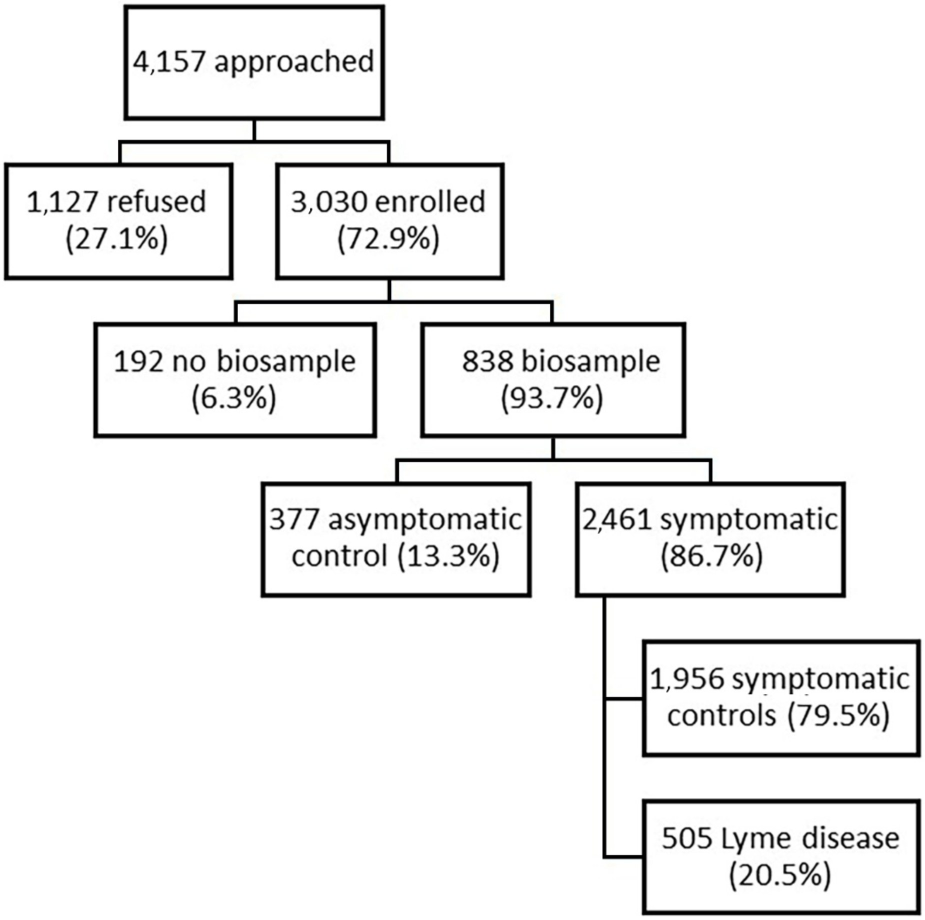

Appendix Figure 2. Flowchart showing characteristics of Pedi Lyme Net study patients, United States, June 2015– March 2020.
